# One Health Approach to Zoonotic Parasites: Molecular Detection of Intestinal Protozoans in an Urban Population of Norway Rats, *Rattus norvegicus*, in Barcelona, Spain

**DOI:** 10.3390/pathogens10030311

**Published:** 2021-03-07

**Authors:** María Teresa Galán-Puchades, María Trelis, Sandra Sáez-Durán, Susana Cifre, Carla Gosálvez, Joan Sanxis-Furió, Jordi Pascual, Rubén Bueno-Marí, Sandra Franco, Víctor Peracho, Tomás Montalvo, Màrius Vicent Fuentes

**Affiliations:** 1 Parasite & Health Research Group, Department of Pharmacy, Pharmaceutical Technology and Parasitology, Faculty of Pharmacy, University of Valencia, Burjassot, 46100 Valencia, Spain; maria.trelis@uv.es (M.T.); sandra.saez@uv.es (S.S.-D.); sue.cimar@gmail.com (S.C.); carla.go.can@gmail.com (C.G.); joansanfu@gmail.com (J.S.-F.); ruben.bueno@uv.es (R.B.-M.); mario.v.fuentes@uv.es (M.V.F.); 2 Department of Research and Development, Laboratorios Lokímica, Paterna, 46980 Valencia, Spain; 3 Pest Surveillance and Control, Agència de Salut Pública de Barcelona (ASPB), 08023 Barcelona, Spain; jpascualsala@gmail.com (J.P.); sfranco@aspb.cat (S.F.); vperacho@aspb.cat (V.P.); tmontal@aspb.cat (T.M.); 4 CIBERESP Epidemiology and Public Health, 08023 Barcelona, Spain

**Keywords:** One Health approach, *Rattus norvegicus*, zoonoses, *Blastocystis*, *Giardia duodenalis*, *Cryptosporidium* spp., *Dientamoeba fragilis*, Barcelona

## Abstract

*Rattus norvegicus*, the brown or Norway rat, is the most abundant mammal after humans in urban areas, where they live in close proximity to people. Among rodent-borne diseases, the reservoir role of Norway rats of zoonotic parasites in cities has practically been ignored. Considering the parasitic diseases in the One Health approach, we intended to identify and quantify the zoonotic intestinal protozoans (ZIP) in an urban population of *R. norvegicus* in the city of Barcelona, Spain. We studied the presence of ZIP in 100 rats trapped in parks (*n* = 15) as well as in the city’s sewage system (*n* = 85) in the winter of 2016/17. The protozoans were molecularly identified by means of a multiplex PCR (Allplex^TM^ Gastrointestinal Panel-Parasite Assay). We also investigated the presence of co-infections among the species found. Four ZIP were identified, presenting significant prevalences in sewers, specifically *Blastocystis* (83.5%)*, Giardia duodenalis* (37.7%), *Cryptosporidium* spp. (34.1%), and *Dientamoeba fragilis* (14.1%). Several co-infections among the detected ZIP were also detected. The reservoir role of ZIP that Norway rats play in cities as well as the role rats may play as sentinels of zoonotic parasites affecting humans in urban areas are strongly backed up by our findings. The increasing worldwide urbanization, climate change, and the COVID-19 pandemic are factors that are producing an increase in human–rat interactions. Our results should be considered a warning to the authorities to intensify rat control and surveillance in public health interventions.

## 1. Introduction

Rodents are hosts of a wide range of zoonotic pathogens. Zoonotic diseases can be transmitted to humans either directly, i.e., the infective agent contaminates the environment and, consequently, humans may become infected due to contaminated hands, food, water, or indirectly through the intervention of an arthropod vector that has become infected directly by the rodent. Among the diseases transmitted by rodents, those caused by bacteria or viruses tend to receive more attention than those caused by parasites (www.cdc.gov/rodents/diseases/index.html (accessed on 15 October 2020)).

The role of rodents as potential reservoirs of zoonotic parasites in rural or wild areas is well known. These can either be protozoans (*Entamoeba histolytica, Giardia duodenalis, Blastocystis* sp., *Cryptosporidium* spp., *Isospora* spp., *Leishmania* spp., *Toxoplasma gondii, Trypanosoma cruzi, Babesia* spp.), helminths (*Brachylaima* spp., *Fasciola hepatica, Schistosoma* spp., *Echinostoma* spp., *Taenia taeniaeformis, Echinococcus* spp., *Hymenolepis* -syn. *Rodentolepis*- *nana*, *H. diminuta*, *Calodium hepaticum* -syn. *Capillaria hepatica*-, *Trichinella spiralis*, *Angisotrongylus cantonensis*, *A. costaricensis, Gongylonema* spp.) or acanthocephalans (*Moniliformis moniliformis*) [1,2].

Among synanthropic rodents, *Rattus norvegicus*, the brown or Norway rat, with a worldwide distribution, is the most commensal species, since its main habitat is always linked to humans. Therefore, people living in close proximity to rodent populations are potentially exposed to infection. To develop a thorough and modern understanding of rat-associated zoonoses, particularly in urban centers, it is crucial to consider the unprecedented rate of global urbanization [3]. Likewise, rodent populations are affected by the climate. In particular, in recent years it has been observed that the rodent populations have increased due to warm, wet winters and springs [4]. Besides climate change, the COVID-19 pandemic has led to an increase in rat sightings in urban slums that may, in turn, increase residents’ exposure and susceptibility to zoonotic diseases transmitted by these rat populations [5].

However, and concerning rat-borne zoonoses, worldwide, but particularly in Europe, data on zoonotic parasites in Norway rats from urban areas (where sewer rats are the most abundant mammal after humans) are scarce and practically absent in the case of intestinal zoonotic protozoans since the vast majority of these studies in *R. norvegicus* have been carried out in rural or wild environments [2,3,6,7].

The term “One Medicine” was coined in 1984 to integrate human and veterinary health and research. The “One Health” concept was introduced at the beginning of the 21st century, emphasizing that human and animal health are interdependent and connected to the ecosystems in which they exist. One of the areas in which the One Health approach is particularly relevant is in the control of zoonoses.

Nonetheless, most of the components of this One Health approach are frequently ignored in zoonotic diseases in which only one aspect is often tackled, i.e., the parasite in question is exclusively considered in humans. This is akin to treating the outward symptoms of a disease but not the underlying cause [8]. Providing accurate and timely information about the parasite in humans and animal reservoirs integrated in the environment that they share is a key element to an effective response to a zoonotic disease threat. To reduce the risk of infection, identifying the pathways by which parasites may spread between animals and humans is essential [9].

Following this One Health concept, the aim of this article is to identify and quantify zoonotic intestinal protozoans (ZIP) and possible co-infections among them in an urban population of *R. norvegicus* in the city of Barcelona, Spain, to which humans are exposed.

It has been calculated that, only considering the sewage system, there are about 0.16 rats/person in the city of Barcelona [10]. The estimated population in Barcelona in 2019 was 1,636,762 inhabitants (https://www.idescat.cat/emex/?id=080193&lang=es (accessed on 2 November 2020)). Consequently, about 262,000 rats circulate in Barcelona’s sewage system. Knowing approximately the number of rats in sewers will also allow calculating the approximate number of infected rats with each ZIP.

## 2. Methods

### 2.1. Study Area and Hosts

One hundred *R. norvegicus* (49 males and 51 females; 67 adults and 33 juveniles) were examined in order to identify their intestinal protozoan parasites. Eighty-five rats were caught at 19 different locations in the sewage system (maximum 10 rats per location) and 15 rats were caught in 7 different parks, covering 8 out of the 10 different districts of the city of Barcelona [11]. In the winter of 2016/17, snap traps were used in the sewage system, whilst wire rat cages were located in parks. Live animals were euthanized by exposure to a CO_2_-saturated atmosphere. Viscera of the trapped rats were preserved in 85% ethanol until their posterior study.

### 2.2. Molecular Techniques

The intestinal content of the rats was collected and kept in 70% ethanol until its study. The samples were filtered and concentrated by centrifugation (2500 rpm, 5 min) in Midi Parasep^®^ SF (Apacor Ltd., Wokingham, UK). This technique is a solvent-free fecal parasite concentrator that is without ether or ethyl acetate for the clean and efficient concentration of helminth eggs, larvae, protozoan cysts, and oocysts.

A part of the concentrated sample, destined for the extraction of stool DNA for PCR testing, was kept at −80 °C. DNA was extracted from 200 µL of the fecal concentrate with the QIAamp DNA Stool Mini Kit (QIAGEN^®^, Hilden, Germany) according to the manufacturer’s instructions.

Multiplex PCR for the detection and identification of intestinal parasites was performed with the Allplex^TM^ Gastrointestinal Panel-Parasite Assay (GIPPA) (Seegene Inc., Seoul, Korea) [12,13]. This panel consists of a multiplex real time PCR for the detection of human protist parasites such as *Giardia duodenalis*, *Entamoeba histolytica*, *Cryptosporidium* spp., *Blastocystis hominis*, *Dientamoeba fragilis,* and *Cyclospora cayetanensis* by amplifying small subunit ribosomal RNA (SSU rRNA) genes. Amplification was performed on the CFX96^TM^ Real Time PCR System (Bio-Rad, Marnes-la-Coquette, France), and managed with CFX Manager IVD 1.6 software, in a 25 µL reaction volume containing 20 µL reaction mix (5 µL Primers Mom, 5 µL Anyplex PCR MM (EM2), 8 µL of water without RNA and 2 µL of internal control and 5 µL of DNA. Negative (DNase/RNase-free water) and positive controls were included in each assay. All PCR runs included both positive and negative controls. Results were analyzed using the Seegene Viewer V2.1 software optimized for multiplex assays. Samples were considered positive for specific parasites if the cycle threshold (Ct) was ≤43 cycles according to the manufacturer’s instructions.

*Blastocystis, G. duodenalis*, and *Cryptosporidium* spp. isolates that tested positive by qPCR were subsequently analyzed for sequencing and genotyping/sub-genotyping. In the case of *Blastocystis*, a direct PCR assay was used targeting the SSU rRNA. The multilocus genotyping (MLG) approach, based on sequencing data generated by PCR of the genes glutamate dehydrogenase (GDH) and ß-giardin (BG), was used for *G. duodenalis*. *Cryptosporidium* spp.-positive isolates were analyzed at the GP60 locus and the SSU marker.

In no case was it possible to obtain typeable sequences of any parasite. The limited sensitivity of conventional PCR assays was a direct consequence of the low concentration of parasitic DNA available as template in their respective amplification reactions. This problem was probably exacerbated by the loss of DNA quality due to the fact that the rats had been stored in ethanol for several months from their capture until the molecular analysis of their intestinal content was carried out.

### 2.3. Statistical Methods

The number of infected rats, as well as the prevalence of each ZIP species were analyzed. Standard non-parametric tests were applied to analyze the influence of rat sex and age on the ZIP prevalence by Binary Logistic Regression (BRL). Positive ZIP associations were calculated by means of a chi-squared test (χ^2^). Statistical significance was established at *p* < 0.05. The IBM SPSS Statistics 26 (IBM Corporation, Amonk, New York, NY, USA) for Windows software package was used for statistical analysis.

## 3. Results

The molecular analysis of the 100 Norway rats revealed the presence of the following ZIP: *Blastocystis, Giardia duodenalis*, *Cryptosporidium* spp., and *Dientamoeba fragilis*. Neither *Entamoeba histolytica* nor *Cyclospora cayetanensis* were detected. Table S1 shows the Allplex^TM^ GI-parasites Assay multiplex PCR results in the positive rats.

Table 1 details the ZIP prevalences overall and by species, both in all the studied rats as well as according to the trapping area. Rats captured in the sewage system did not only have a higher overall ZIP prevalence than those captured in parks (87.1% vs. 53.3%, χ^2^ = 7.673; *p* = 0.0056), but also of each one of all the protozoan species, statistically significant in the cases of *Blastocystis* and *Cryptosporidium* spp. (χ^2^ = 11.294; *p* = 0.0008 and χ^2^ = 5.646; *p* = 0.0175, respectively). However, the smaller number of rats captured in parks compared to those captured in sewers should be considered (15 vs. 85).

The statistical comparison between sex and age groups did not reveal any differences neither in the overall prevalence of ZIP, nor in the prevalence of any particular species between males and females or between juveniles and adults.

In relation to co-infections, 43.9% of the infected rats harbored only one ZIP. Additionally, 29.3% were infected by two species, 23.2% with three, and 3.7% were infected with the four ZIP detected. These percentages slightly increase in rats from the sewage system (47.3% with one species, 35.1% with two, 29.7% with three, and 5.4% with the four ZIP). The statistically significant co-infections found were: 32% *Blastocystis*-*G. duodenalis* (χ^2^ = 5.138; *p* = 0.0234); 28% *Blastocystis*-*Cryptosporidium* spp. (χ^2^ = 7.330; *p* = 0.0068); and 18% *G. duodenalis*-*Cryptosporidium* spp. (χ^2^ = 4.581; *p* = 0.0323).

## 4. Discussion

Concerning data on ZIP in urban populations of *R. norvegicus* around the world, as far as we know, only *Cryptosporidium* spp. has been molecularly identified in urban areas of Nishinomiya City (Japan), Tehran (Iran), and New York (USA) [14,15,16] and *Giardia* spp. also in Iran [15]. Likewise, *Cryptosporidium* oocysts and *Giardia* cysts have morphologically been determined in the city of Buenos Aires (Argentina) [17]. *Blastocystis* cysts were morphologically identified in brown rats in Kuala Lumpur (Malaysia) [18]. In Europe, apparently, the only ZIP identified in cities in the Norway rat has been the microsporidian species *Encephalitozoon cuniculi* that was found in Zurich (Switzerland) [19].

All ZIP were found in the rats captured in the sewage system. However, *Cryptosporidium* spp. was not found in those trapped in parks, although, as already specified, the lower number of rats studied in parks should be taken into account. Regarding the overall prevalence of ZIP, 82 of the 100 analyzed rats harbored at least one ZIP species, which evidences that they are broadly distributed in the Barcelona rat population without sex or age distinction.

All the ZIP identified have direct or monoxenous life cycles that follow the same fecal-oral transmission route. Therefore, the infective parasite forms of human *Blastocystis, G. duodenalis,* and *D. fragilis* (cysts) and *Cryptosporidium* spp. (oocysts) are shed in feces that contaminate the city’s sewage system in which large populations of *R. norvegicus* live. These common ZIP life cycles are among the reasons that may explain why co-infections have been found in the rats under study.

### 4.1. Blastocystis

The majority of *Blastocystis* parasites does not have specific hosts [20]. It has been calculated that one billion people are infected by *Blastocystis* although its public health significance remains unknown. Few studies have investigated the prevalence of *Blastocystis* infection in Spain. However, particularly in Barcelona, a retrospective observational study was carried out at the Vall d’Hebron University Hospital between 2009–2014 [21]. 418 cases were diagnosed, 22% of them being symptomatic, suggesting that *Blastocystis* is pathogenic at least under specific circumstances.

As already pointed out, the only known data of *Blastocystis* infection in urban Norway rats so far was obtained in Kuala Lumpur (Malaysia), where a prevalence of 51% was found [18]. Our study reveals a prevalence of 83.5% in rats from sewers, the highest prevalence among ZIP. That means, considering the number of Norway rats in sewers (about 262,000), that there are more than 218,000 rats infected by *Blastocystis* only in the sewage system.

The zoonotic contribution to human *Blastocystis* colonization is unknown although it has been considered low [22]. However, the obtained results strongly suggest the role of *R. norvegicus* as a source of human blastocystiasis infection.

### 4.2. Giardia duodenalis

A *G. duodenalis* prevalence of 37.7% was found in sewers, which means that there probably are about 99,000 infected rats with this species circulating in Barcelona’s sewage system. The only other molecular study on *Giardia* spp. in urban brown rats recently carried out in Teheran (Iran) revealed a higher prevalence of 76%, although considering that it was a generic identification other *Giardia* spp. from rodents could have also been involved [15].

As aforementioned, it was not possible to determine *Giardia* genetic assemblages in this study. Of the eight currently recognized assemblages (A to H), only A and B correspond to humans [23]. To date, only the non-human assemblage G, typical of rodents, has been found in rural Norway rats in China [24]. However, the human assemblage B was detected in the black rat, *R. rattus*, in an inhabited area of La Palma Island (Canary Islands, Spain) [25]. The finding of assemblages other than the rodent-specific assemblage G in rodents may be due to the habitat shared by wildlife with domestic animals and humans [26]. Norway rats in cities, specifically those living in the sewage system, are in permanent close contact with human fecal disposal. Consequently, the same assemblages that infect humans are likely to infect rats. In Barcelona, both assemblages A and B have been identified in humans [27]. Therefore, and considering the high number of *Giardia*-infected rats found (although confirmative genetic studies are necessary), the One Health approach in giardiasis should include not only the potential reservoir role of companion animals [28], but also that of brown rats in cities.

### 4.3. Cryptosporidium spp.

Cryptosporidiasis is one of the main causes of diarrhea both in humans and animals worldwide. Specifically in Spain, the number of cases of cryptosporidiasis increased by 175% in 2018 with respect to those of 2017 (www.isciii.es (accessed on 10 November 2020)). To tackle the disease with a One Health approach, a better understanding of the zoonotic transmission is essential.

Although two species, *Cryptosporidium hominis* and *C. parvum,* cause 90% of the infection in humans, 19 other species have also been reported to infect humans [29]. A vast number of mammals act as reservoirs including various rodents, bovids, camelids, equids, canids, felids, rabbits, etc. [30]. However, without the assistance of advanced molecular methods it is not possible to ascertain the species/genotypes involved. That is why initial findings of *Cryptosporidium* oocysts in mammals other than humans were assumed to be *C. parvum.*

Rural *R. norvegicus* was considered reservoir for *C. parvum* [30]. However, the study was based only on the morphology of the shed oocysts. Genetic studies in wild rodents in Europe revealed the presence, among others, of *C. hominis* and *C. parvum.* Norway rats were negative in the survey although only four individuals were studied [31].

Molecular studies carried out in urban *R. norvegicus* in Nishinomiya, Tehran and New York revealed *Cryptosporidium* prevalences of 38%, 1% and 1.5%, respectively [13,14,15]. So far, only the zoonotic species *C. muris* and C. *occultus* have been genetically identified in wild brown rats in China, and the latter also in the Czech Republic [32,33].

In our study, *Cryptosporidium* spp. was only found in rats from sewers with practically the same prevalence as that found in Japan (37.7% vs. 38%). Following its prevalence (Table 1), there are more than 89,000 infected rats in sewers in Barcelona. The same as in the case of giardiasis, although further genetic studies are required, *R. norvegicus* is a clear potential reservoir of human cryptosporidiasis in cities.

### 4.4. Dientamoeba fragilis

*Dientamoeba fragilis* is considered a neglected intestinal protozoan [34]. It is reported worldwide, causing human gastrointestinal symptoms, and it is considered the second most common intestinal protozoan after *Blastocystis,* with an incidence even higher than *Giardia duodenalis*. In Barcelona, cases have been described in immigrant patients, as well as in autochthonous citizens, and travelers [35].

Two major *D. fragilis* genotypes have been described, but the overall significance with regard to pathogenicity is unclear [36]. Other than humans, few animal hosts of *D. fragilis* have been reported (gorillas, macaques, baboons, and pigs) [36]. However, considering the worldwide pig population and the closer contact with humans, only pigs are considered reservoirs of *D. fragilis* [37]. Hitherto, rodents have only been experimental hosts [38].

We found *D. fragilis*-infected Norway rats both in parks (6.7%) and in the sewage system (14.1%), which means the presence of infected rats in sewers in Barcelona is about 37,000. This is the first time that naturally infected *R. norvegicus* by *D. fragilis* have been reported in the world. The Norway rat should therefore be considered a clear potential dientamoebiasis reservoir for the same reasons that pigs are, i.e., due to their large populations and proximity to humans.

### 4.5. Other Zoonotic Parasites

In addition to the found ZIP in the 100 examined rats, previous studies revealed that these same rats were infected by six additional zoonotic parasites, specifically, the protozoan *Leishmania infantum* (33.3% in sewers), the intestinal tapeworms *Hymenolepis diminuta* (33%) and *H. nana* (17%), the hepatic nematode *Capillaria hepatica* (17%), the oesophagic nematode *Gongylonema neoplasticum* (20%), and the intestinal acanthocephalan *Moniliformis moniliformis* (6%) [11,39]. Considering all the zoonotic parasites, protozoans and helminths, 95% of the rats were infected with at least one zoonotic species, i.e., almost 249,000 Norway rats carry some zoonotic parasite in the sewers of the studied city.

## 5. Conclusions

This study reveals by molecular methods, and for the first time, significant *Blastocystis, Giardia duodenalis, Cryptosporidium* spp., and *D. fragilis* prevalences, as well as the occurrence of multiple ZIP co-infections in a population of *R. norvegicus* in an urban environment. Likewise, the Norway rat was found to be a new host for *D. fragilis* and, therefore, a new potential reservoir.

*R. norvegicus* should be considered an (undesirable) effective reservoir of ZIP in urban areas for the following reasons: (i) its population in cities is the largest after that of humans; (ii) in absence of competitors or predators rats live longer in cities (up to three years) than in the wild (up to one year); (iii) the high ZIP prevalences found, specifically in rats from sewers; (iv) Norway rats in cities live in proximity to humans resulting in close interaction; (v) large rat populations live in sewers in close contact with human fecal disposal containing infective parasite forms (cysts, oocysts); (vi) it is not possible to control the environmental contamination by rat feces; and (vii) a proper management of rat fecal waste is also unfeasible.

According to the results obtained, brown rats have been revealed as sensitive indicators of parasitic environmental hazards to humans. Therefore, Norway rats in cities may serve as sentinel species to detect zoonotic parasites affecting humans.

We are confident that this study, as well as the two previous ones on *R. norvegicus* in Barcelona, will be key in considering, integrated in the context of the One Health approach, the involvement of Norway rats in the transmission of zoonotic parasites. These results should also be considered a warning for the authorities to intensify rat control and surveillance in public health interventions.

## Figures and Tables

**Table 1:** Prevalences of overall ZIP, and according to species, in the total of the rats studied, and depending on the trapping area, in *R. norvegicus*, Barcelona, winter 2016–2017.

	Total (*n* = 100)	Parks (*n* = 15)		Sewers (*n* = 85)	
Protozoan species	N	N	%	N	%
*Blastocystis*	77	6	40.0	71	83.5
*Giardia duodenalis*	35	3	20.0	32	37.7
*Cryptosporidium* spp.	29	–	–	29	34.1
*Dientamoeba fragilis*	13	1	6.7	12	14.1
Overall ZIP	82	8	53.3	74	87.1

Abbreviations: *n*, *n*° of animal analyzed; N, *n*° of positive samples; %, prevalence.

## Data Availability

The data presented in this study are available on request from the corresponding author.

## Supplementary Materials

The following are available online at https://www.mdpi.com/2076-0817/10/3/311/s1, Table S1: Allplex^TM^ GI-parasites Assay multiplex PCR results for the 82 positive rats analysed.

## References

[1] Pan American Health Organization (2003). Zoonoses and Communicable Diseases Common to Man and Animals: Parasitoses.

[2] Meerburg B.G., Singleton G.R., Kijlstra A. (2009). Rodent-borne diseases and their risks for public health. Crit. Rev. Microbiol..

[3] Himsworth C.G., Parsons K.L., Jardine C., Patrick D.M. (2013). Rats, cities, people, and pathogens: A systematic review and narrative synthesis of literature regarding the ecology of rat-associated zoonoses in urban centers. Vector Borne Zoonotic Dis..

[4] Semenza J.C., Menne B. (2009). Climate change and infectious diseases in Europe. Lancet Infect. Dis..

[5] Souza F.N., Awoniyi A.M., Palma F.A.G., Begon M., Costa F. (2020). Increased rat sightings in urban slums during the COVID-19 pandemic and the risk for rat-borne zoonoses. Vector Borne Zoonotic Dis..

[6] Mustapha T., Daskum A.M., Majid R.A., Unyah N.Z. (2019). A Review on Rodent-borne Parasitic Zoonosis: Public Health Risk to Humans. South Asian J. Parasitol..

[7] Strand T.M., Lundkvist A. (2019). Rat-borne diseases at the horizon. A systematic review on infectious agents carried by rats in Europe 1995–2016. Infect. Ecol. Epidemiol..

[8] Van Helden P.D., van Helden L.S., Hoal E.G. (2013). One world, one health. EMBO Rep..

[9] World Health Organization, Food and Agriculture Organization of the United Nations & World Organisation for Animal Health (2019). Taking a Multisectoral, One Health Approach: A Tripartite Guide to Addressing Zoonotic Diseases in Countries.

[10] Pascual J., Franco S., Bueno-Marí R., Peracho V., Montalvo T. (2020). Demography and ecology of Norway rats, *Rattus norvegicus,* in the sewer system of Barcelona (Catalonia, Spain). J. Pest. Sci..

[11] Galán-Puchades M.T., Sanxis-Furió J., Pascual J., Bueno-Marí R., Franco S., Peracho V., Montalvo T., Fuentes M.V. (2018). First survey on zoonotic helminthosis in urban brown rats (*Rattus norvegicus*) in Spain and associated public health considerations. Vet. Parasitol..

[12] Kim B., Kim H.S., Kim J.J., Park Y.J., Kim D., Yong D. (2020). Detection of intestinal protozoa in Korean patients using BD MAX Enteric Parasite Panel and Seegene Allplex Gastrointestinal Parasite Assay. Lab. Med. Online.

[13] Autier B., Gangneux J.P., Robert-Gangneux F. (2020). Evaluation of the Allplex^TM^ Gastrointestinal Panel—Parasite Assay for Protozoa Detection in Stool Samples: A Retrospective and Prospective Study. Microorganisms.

[14] Kimura A., Edagawa A., Okada K., Takimoto A., Yonesho S., Karanis P. (2007). Detection and genotyping of *Cryptosporidium* from brown rats (*Rattus norvegicus*) captured in an urban area of Japan. Parasitol. Res..

[15] Azimi T., Pourmand M.R., Fallah F., Karimi A., Mansour-Ghanaie R., Hoseini-Alfatemi S.M., Shirdoust M., Azimi L. (2020). Serosurvey and molecular detection of the main zoonotic parasites carried by commensal *Rattus norvegicus* population in Tehran, Iran. Trop. Med. Health.

[16] Firth C., Bhat M., Firth M.A., Williams S.H., Frye M.J., Simmonds P., Conte J.M., Ng J., García J., Bhuva N.P. (2014). Detection of zoonotic pathogens and characterization of novel viruses carried by commensal *Rattus norvegicus* in New York. mBio.

[17] Hancke D., Suárez O.V. (2020). Co-occurrence of and risk factors for *Cryptosporidium* and *Giardia* in brown rats from Buenos Aires, Argentina. Zoonoses Public Health.

[18] Premaalatha B., Chandrawathani P., Farah Haziqah M.T., Jamnah O., Zaini C.M., Ramlan M. (2017). A survey of endoparasite and ectoparasite infections of wild rats caught in areas of Ipoh and Kuala Lumpur, Malaysia. Malays. J. Vet. Res..

[19] Müller-Doblies U.U., Herzog K., Tanner I. (2002). First isolation and characterisation of *Encephalitozoon cuniculi* from a free-ranging rat (*Rattus norvegicus*). Vet. Parasitol..

[20] Adl S.M., Bass D., Lane C.E., Lukes J., Schoch C.L., Smimov A., Agatha S., Berney C., Brown M.W., Burki F. (2019). Revisions to the Classification, Nomenclature, and Diversity of Eukaryotes. J. Eukaryot. Microbiol..

[21] Salvador F., Sulleiro E., Sánchez-Montalvá A., Alonso C., Santos J., Fuentes I., Molina I. (2016). Epidemiological and clinical profile of adult patients with *Blastocystis* sp. infection in Barcelona, Spain. Parasit. Vectors.

[22] Stensvold C.R., Tan K.S.W., Clark C.G. (2020). Blastocystis. Trends Parasitol..

[23] Heyworth M. (2016). *Giardia duodenalis* genetic assemblages and hosts. Parasite.

[24] Zhao Z., Wang R., Zhao W., Qi M., Zhao J., Zhang L., Li J., Liu A. (2015). Genotyping and subtyping of *Giardia* and *Cryptosporidium* isolates from commensal rodents in China. Parasitology.

[25] Fernández-Álvarez A., Martín-Alonso A., Abreu-Acosta N., Feliu C., Hugot J.P., Valladares B., Foronda P. (2014). Identification of a novel assemblage G subgenotype and a zoonotic assemblage B in rodent isolates of *Giardia duodenalis* in the Canary Islands, Spain. Parasitology.

[26] Feng Y., Xiao L. (2011). Zoonotic potential and molecular epidemiology of *Giardia* species and Giardiasis. Clin. Microbiol. Rev..

[27] Wang Y., Gonzaález-Moreno O., Roellig D.M., Oliver L., Huguet J., Guo Y., Feng Y., Xiao L. (2019). Epidemiological distribution of genotypes of *Giardia duodenalis* in humans in Spain. Parasit. Vectors.

[28] Krecek R.C., Rabinowitz P.M., Conrad P.A. (2020). Demystifying and demonstrating the value of a One Health approach to parasitological challenges. Vet. Parasitol..

[29] Feng Y., Ryan U.M., Xiao L. (2018). Genetic diversity and population structure of *Cryptosporidium*. Trends Parasitol..

[30] Quy R.J., Cowan D.P., Haynes P.J., Sturdee A.P., Chalmers R.M., Bodley-Tickell A.T., Bull S. (1999). AThe Norway rat as a reservoir host of *Cryptosporidium parvum*. J. Wilf. Dis..

[31] Danišová O., Valenčáková A., Stanko M., Luptáková L., Hatalová E., Čanády A. (2017). Rodents as a reservoir of infection caused by multiple zoonotic species/genotypes of *C. parvum, C. hominis, C. suis, C. scrofarum*, and the first evidence of *C. muskrat* genotypes I and II of rodents in Europe. Acta Trop..

[32] Kvác M., Vlnatá G., Jezcová J., Horcicková M., Konecný R., Hlásková L., McEvoy J., Sak B. (2018). *Cryptosporidium occultus* sp. n. (Apicomplexa: Cryptosporidiidae) in rats. Eur. J. Protist..

[33] Zhao W., Zhou H., Huang Y., Xu L., Rao L., Wang S., Wang W., Yi Y., Zhou X., Wu Y. (2019). *Cryptosporidium* spp. in wild rats (*Rattus* spp.) from the Hainan Province, China: Molecular detection, species/genotype identification and implications for public health. IJP PAW.

[34] García L.S. (2016). *Dientamoeba fragilis*, One of the Neglected Intestinal Protozoa. J. Clin. Microbiol..

[35] Miguel L., Salvador F., Sulleiro E., Sánchez-Montalvá A., Molina-Morant D., Lopez I., Molina I. (2018). Clinical and epidemiological characteristics of patients with *Dientamoeba fragilis* infection. Am. J. Trop. Med. Hyg..

[36] Van Gestel R.S.F.E., Kusters J.G., Monkelbaan J.F. (2018). A clinical guideline on *Dientamoeba fragilis* infections. Parasitology.

[37] Cacciò S.M., Sannella A.R., Manuali E., Tosini F., Sensi M., Crotti D., Pozio E. (2012). Pigs as natural hosts of *Dientamoeba fragilis* genotypes found in humans. Emerg. Infect. Dis..

[38] Munashingue V.S., Vella N.G.F., Ellis J.T., Windsor P.A., Stark D. (2013). Cyst formation and faecal–oral transmission of *Dientamoeba fragilis*—The missing link in the life cycle of an emerging pathogen. Int. J. Parasitol..

[39] Galán-Puchades M.T., Gómez-Samblás M., Suárez-Morán J.M., Osuna A., Sanxis-Furió J., Pascual J., Bueno-Marí R., Franco S., Peracho V., Montalvo T. (2019). Leishmaniasis in Norway rats in sewers, Barcelona, Spain. Emerg. Infect. Dis..

