# Supplementary material for: One Health Approach to Zoonotic Parasites: Molecular Detection of Intestinal Protozoans in an Urban Population of Norway Rats, Rattus norvegicus, in Barcelona, Spain
Source: Pathogens. 2021 Mar 7;10(3):311. doi: 10.3390/pathogens10030311 (PMC7998591; doi:10.3390/pathogens10030311)
Supplement: Supplementary file 1 [file pathogens-10-00311-s001.pdf]

Table S1. Allplex™ GI-parasites Assay multiplex PCR results for the 82 positive rats analysed. *Bh, Blastocystis*; *Gd, Giardia duodenalis*; *Df, Dientamorba fragilis*; *Cr, Cryptosporidium*. Ct, cycle threshold. Cutoff established: positive result, C_t <43; negative result, C_t ≥43. C-, negative control; C+, positive control.

Rat nº	*Bh* Ct	*Gd* Ct	*Df* Ct	*Cr* Ct
1	37.31	-	-	37.42
2	31.27	35.52	38.45	35.70
3	37.23	38.01	-	38.93
4	36.38	-	-	-
5	39.73	38.14	-	-
7	-	39.32	-	-
8	-	38.68	-	-
9	34.22	38.29	-	40.19
10	38.30	37.80	-	-
11	-	-	35.59	-
12	36.19	37.93	36.43	39.88
13	38.07	-	-	-
14	34.63	38.13	37.74	-
15	36.08	35.65	40.41	38.27
16	35.72	-	39.76	-
17	37.06	-	-	-
18	35.26	38.92	38.94	-
19	38.37	-	-	-
22	38.89	-	-	-
23	39.29	-	-	-
24	38.11	-	-	-
25	38.88	-	-	-
27	39.72	-	-	-
28	40.94	-	-	-
29	39.99	38.74	-	-
31	32.92	34.36	-	40.31
32	-	-	-	39.98
33	39.76	38.89	-	-
34	39.57	-	-	-
35	38.83	-	-	38.22
36	38.04	38.40	-	36.25
37	31.46	-	-	-
38	39.93	-	-	-
39	38.08	39.80	-	-
40	39.02	-	-	-
41	34.22	-	40.92	40.07
42	37.54	36.81	-	-
43	37.56	-	-	-
45	33.75	35.65	-	37.11
46	34.45	36.82	-	37.58
47	31.34	-	39.69	-
48	37.79	-	-	-
49	30.57	37.44	-	-
50	25.29	40.07	-	39.62

51	34.25	32.55	40.83	-
52	39.95	-	-	36.76
53	30.33	-	-	-
54	35.05	-	39.29	-
55	38.94	-	-	-
56	34.20	38.73	-	40.07
57	39.72	-	-	-
58	36.68	-	-	38.55
61	40.65	-	-	-
64	37.37	39.46	-	-
65	37.65	38.38	-	39.98
68	39.21	40.13	-	39.82
69	38.97	-	-	39.16
70	40.46	-	-	-
71	36.00	-	-	-
72	40.24	39.43	-	-
73	36.76	32.42	-	38.84
75	37.17	38.19	-	35.21
76	39.71	-	38.38	-
79	38.83	39.95	-	-
80	29.39	-	-	38.04
81	-	37.47	-	-
82	38.84	-	-	36.71
83	39.17	-	-	33.05
84	32.76	36.57	-	39.43
85	33.93	-	-	38.32
86	37.13	-	-	-
88	35.59	39.82	-	-
89	39.35	-	-	-
91	36.15	-	-	-
92	37.25	-	-	-
94	31.51	38.72	-	37.54
95	36.24	-	-	-
97	39.75	-	-	-
98	31.48	36.17	-	37.77
99	29.52	-	37.32	-
101	39.01	-	-	-
102	38.10	-	-	-
**C-**	**-**	**-**	**-**	**-**
**C+**	**25.98**	**20.56**	**25.78**	**23.00**
